# A comparison of conventional methods for the quantification of bacterial cells after exposure to metal oxide nanoparticles

**DOI:** 10.1186/s12866-014-0222-6

**Published:** 2014-08-21

**Authors:** Hongmiao Pan, Yongbin Zhang, Gui-Xin He, Namrata Katagori, Huizhong Chen

**Affiliations:** 1 Division of Microbiology, National Center for Toxicological Research, U.S. Food and Drug Administration, Jefferson 72079, AR, USA; 2 Nanotechnology Core Facility, National Center for Toxicological Research, U.S. Food and Drug Administration, Jefferson 72079, AR, USA; 3 Department of Clinical Laboratory and Nutritional Sciences, University of Massachusetts Lowell, Lowell 01854, MA, USA

**Keywords:** Nanoparticles, Bacterial quantification, Flow cytometry (FCM), Colony-forming units (CFU), Optical density (OD)

## Abstract

**Background:**

Due to potential interference of nanoparticles on bacterial quantification, there is a challenge to develop a fast, accurate and reproducible method for bacterial quantification. Currently various bacterial quantification methods are used by researchers performing nanoparticles study, but there has been no efficacy evaluation of these methods. Here we study interference of nanoparticles on three most commonly used conventional bacterial quantification methods, including colony counting to determine the colony-forming units (CFU), spectrophotometer method of optical density (OD) measurement, and flow cytometry (FCM).

**Results:**

Three oxide nanoparticles including ZnO, TiO_2_, and SiO_2_ and four bacterial species including *Salmonella enterica* serovar Newport*, Staphylococcus epidermidis*, *Enterococcus faecalis*, and *Escherichia coli* were included in the test. Results showed that there is no apparent interference of the oxide nanoparticles on quantifications of all four bacterial species by FCM measurement; CFU counting is time consuming, less accurate and not suitable for automation; and the spectrophotometer method using OD measurement was the most unreliable method to quantify and detect the bacteria in the presence of the nanoparticles.

**Conclusion:**

In summary, FCM measurement proved to be the best method, which is suitable for rapid, accurate and automatic detection of bacteria in the presence of the nanoparticles.

## Background

Nanoparticles (NPs) offer spectacular properties to their bulk materials, such as a high surface area to volume ratio, new mechanical, chemical, electrical, optical, magnetic, electro-optical, and magneto-optical properties [1]. Nanotechnology is one of the fastest growing areas of the high tech economy [2],[3]. Products using nanoparticles - also known as nanomaterials (particle sizes less than 100 nm)-can be found in almost every area of our daily lives, from cosmetics to clothing to foods to drug products [4]-[7]. There are hundreds of cosmetics that contain nanomaterials, such as ZnO, TiO_2_, and SiO_2_, in the market now and the number of these products are increasing rapidly [8]. Nanoscale materials can find use in many areas related to the food industry including agriculture, food processing, food security, packaging, nutrition and neutraceuticals [9]-[11]. Nanoscale materials have been used as novel antimicrobial agents [12]. Due to their powerful antimicrobial activity and particular modes of action, nanoparticles provide an attractive alternative to classic antibiotics in the development of next-generation antibiotic agents [13]-[15]. Various antimicrobial nanoparticles and nanosized carriers for antibiotics delivery have been developed to effectively treat infectious diseases, especially those caused by antibiotic-resistant microorganisms [16],[17]. Nanoparticles behave differently from their respective bulk materials and thus the unique properties of the nanoparticles might also cause adverse health effects on human, animal and environment. The speedy commercialization of nanotechnology requires thoughtful and careful environmental, animal and human health safety assessment [18],[19].

The detection and quantification of viable bacteria plays a critical role in quality control programs of the food, cosmetics and drug industry to prevent illness and in clinical diagnosis and therapeutics. Currently there are many methods used for the detection and quantification of bacteria, ncluding conventional and molecular approaches [20]-[24]. Conventionally identification of bacteria is usually performed by three methods including culture-based counting for colony-forming units (CFU) [22],[25], spectrophotometer method of optical density (OD) measurement [23],[24], and flow cytometry (FCM) [26],[27]. In spite of the sensitivity and reliability, counting CFU is time-consuming and labor-intensive [28],[29]. CFU determination is the conventional method to quantify bacteria, but only detects those that are able to grow on specific solid media, which excludes the detection of unculturable live, inactive or damaged bacterial cells [30],[31]. Therefore, CFU counting tends to undercount the actual number of the bacteria. For example, anaerobic bacteria are not able to grow on the media and cultural conditions suitable for growth of aerobic bacteria. Optical density method measures turbidity associated directly with bacterial growth which is rapid, low cost and non-destructive, however, it measures live as well as dead bacterial cell debris. Flow cytometry is a relatively newly developed technique and enables a fast and reliable detection of all bacteria including the non-cultivable microorganisms. It enables researchers to reliably distinguish and quantitate live and dead bacteria with the aid of a flow cytometer in a mixed population containing various bacterial types [32]. Besides, Flow cytometry method is able to provide morphometric and functional properties of the detected bacteria [33],[34]. Currently all these three methods are employed to quantify bacterial contents in the presence of nanoparticles [35]-[39]. So far there has not been any research performed concerning potential interference by nanoparticles on the bacterial counting methods.

The aim of this study was to compare three commonly used conventional methods for bacterial detection and quantification in the presence of nanoparticles. In this study, three nanoparticles, ZnO, TiO_2_, and SiO_2,_ which are commonly used in commercial products and four important human pathogens (*Salmonella enterica* serovar Newport, *Staphylococcus epidermidis*, *Enterococcus faecalis*, and *Escherichia coli*) frequently found in various products and environment representing Gram-positive and Gram-negative bacteria were employed for this study.

## Results and discussion

### Physical and chemical characterizations of nanomaterials

**Figure 1:**
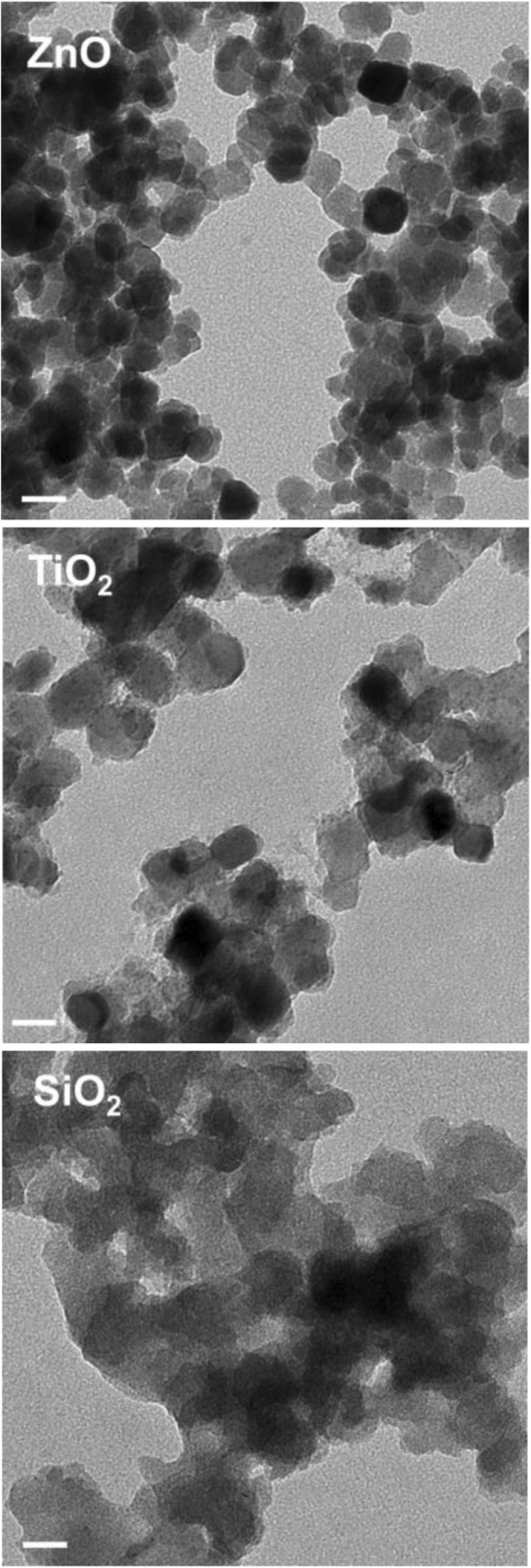
**Characterization of ZnO, TiO**_**2**_**, or SiO**_**2**_**nanoparticles by transmission electron microscopy (TEM).** Nanoparticles were deposited on formvar carbon coated grids and dried for TEM imaging. Images were analyzed in high resolution mode with an acceleration voltage of 100 kV. Morphology of ZnO, TiO2 or SiO_2_ is shown in left, middle and right of the above images. Scale Bar = 20 nm.

It is critical to conduct physical and chemical characterization of testing nanomaterials in nanotechnology research. Size, size distribution, surface charge, aggregation or agglomeration status, and shape have been considered as the most important parameters for nanomaterials. We evaluated these parameters using TEM and Zetasizer as described in the material and methods section. TEM analysis indicated that the ZnO, TiO_2_ and SiO_2_ nanoparticles have spherical shape with slightly agglomeration (Figure 1). The primary size of ZnO, TiO_2_ and SiO_2_ nanoparticles were measured as 14.0 ± 4.9 nm, 19.7 ± 5.7 nm and 17.4 ± 5.1 nm, respectively (Table 1). The range of the diameter of the ZnO, TiO_2_ and SiO_2_ nanoparticle was 6.3-30.5 nm, 10.2-31.2 nm and 8.0-27.9 nm. Zetasizer analysis indicated that the average size of ZnO, TiO_2_ and SiO_2_ nanoparticles in buffer solution was 2308.3 ± 159.1 nm, 2738.3 ± 303.3 nm and 915.0 ± 35.8 nm (mean ± SD). The average surface charge of the ZnO, TiO_2_, SiO_2_ nanoparticles in buffer solution was 17.6 ± 0.7 mV, 27.2 ± 3.1 mV, −5.7 ± 0.4 mV, respectively (Table 1). TEM directly measured the primary size of the nanoparticles based on the projected area; while Dynamic Light Scattering (DLS) measured the hydrodynamic diameter of the nanoparticles based on the translational diffusion area of the particle being measured. The same samples of these nanoparticles in buffer were measured with a bigger size by zetasizer analysis than the measurement using TEM. This is due to the differences in the weighted averages determined by these two techniques, and also the differences in the physical properties measured. TEM is sensitive to the size of primary particles, whereas DLS is sensitive to the presence of small quantities of large particles or aggregates.

**Table 1:** **Characterization of TiO**_**2**_**, ZnO, and SiO**_**2**_**nanoparticles in Milli-Q water solutions**

**Physical Parameters**	**ZnO**	**TiO**_**2**_	**SiO**_**2**_
Primary size (nm)	14.0 ± 4.9	19.7 ± 5.7	17.4 ± 5.1
Primary size range(nm)	6.3 – 30.5	10.2 - 31.2	8.0 – 27.9
Hydrodynamic size (nm)	2738.3 ± 303.3	2308.3 ± 159.1	915.0 ± 35.8
Shape	spherical	spherical	spherical
Agglomerate in solution	Yes	Yes	Yes
Zeta potential ζ (mV)	17.6 ± 0.7	27.2 ± 3.1	−5.7 ± 0.4

### Effect of concentrations of nanoparticle on quantification of bacteria

**Figure 2:**
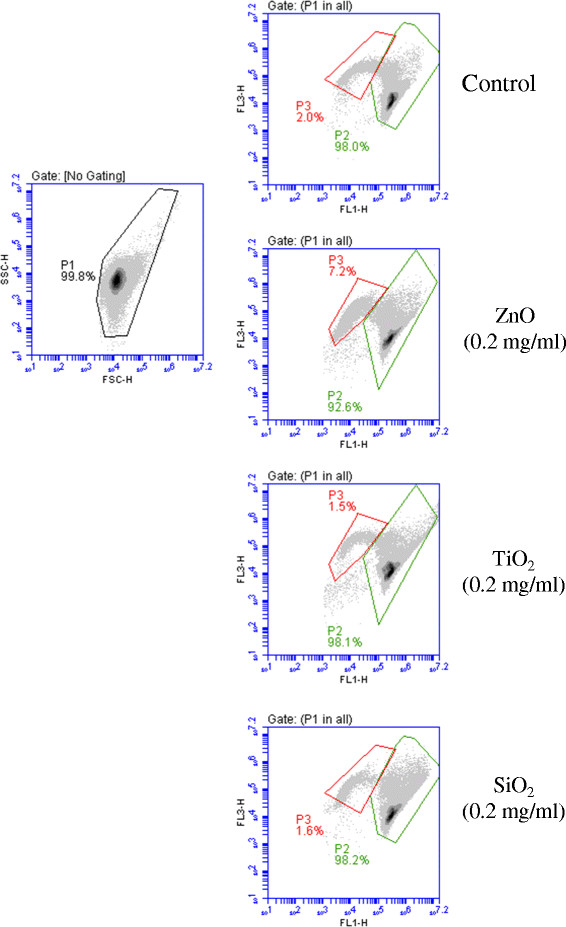
**Examples of flow cytometric for*E. faecalis*exposure to nanoparticles-ZnO, TiO**_**2**_**, and SiO**_**2**_**at concentration of 0.2 mg/ml.** Fluorescence (FL1-H/FL3-H) was tested from bacterial cells inside gate P1 in a FSC-H (forward scatter-H)/SSC-H (side scatter-H) density plots. Live bacterial cells (gate P2); dead bacterial cells (gate P3).

Potential interference of nanoparticles on the quantification of bacteria poses a challenge for the detection of bacterial contaminations in various consumer products. Therefore, development of a rapid, sensitive and accurate method for detection of bacteria in the presence of nanoparticles is crucial for food, drug, cosmetic and other consumable products. Among many bacterial identification and quantification methods, three of them including culture-based counting for CFU, spectrophotometer method of optical density measurement, and more recently flow cytometry are commonly used. ZnO, TiO_2_, and SiO_2_ have been found in many commercial products including food, food supplements, cosmetics and drugs. *S. enterica* Newport, *S. epidermidis*, *E. faecalis*, and *E. coli*, which are important human pathogens, are good representatives for Gram-positive and Gram-negative bacteria (Table 2). In this experiment the effect of various concentrations of nanoparticles on quantification of *S. enterica* Newport, *S. epidermidis*, *E. faecalis*, and *E. coli* was investigated by exposing 5 ml of samples containing approximately 10^9^ cells/ml to various concentrations of ZnO, TiO_2_, and SiO_2_ (0, 0.1, 0.2, 0.3, 0.5, and 1 mg/ml final concentration) for 1 hr, respectively (Table 3). As shown in Table 3, with increasing concentrations of ZnO, TiO_2_, and SiO_2_, there was no apparent interference of the nanoparticles on quantifications of all four bacterial species by flow cytometry measurement using the BacLight LIVE/DEAD bacterial viability and counting kit. As shown in Figure 2 as example, two distinctive groups were formed. Group P2 was the population of living bacterial cells, while group P3 was the population of dead bacterial cells at the presence of 0.2 mg/ml nanoparticles. Compared to a control, which did not contain nanoparticles, no shifts of the bacterial population or background increase were observed (Figure 2). Since more than 20,000 bacterial cells per sample were counted by flow cytometry measurement, high accuracy and excellent reproducibility of the quantification was achieved for both live and dead bacterial cells (Table 3). Although no apparent interference of the nanoparticles on quantifications of all four bacterial species was observed by using CFU counting, it was a time consuming and labor intensive procedure. Besides, it took long time training and practice for mastering the technique of dilution in order to get reliable counts from one batch to another and from one plate to another in CFU counting. Furthermore, the data obtained by CFU measurement is less accurate and reproducible due to a limited number of bacterial cells counted (several hundred bacterial colonies counted (Table 3). The decreasing numbers of the bacteria by using CFU and flow cytometry were resulted from antibacterial effects caused by both nanoparticles TiO_2_ and ZnO. As shown in Table 3, nanoparticles had adverse effect on quantification of bacteria using the spectrophotometer method of optical density measurement with severity of TiO_2_ > ZnO > SiO_2._ For example, in the presence of 0.1 mg TiO_2_, the number of *S. epidermidis* cells was not detectable due to high background interference from the nanoparticles in the samples. With 0.3 mg/ml TiO_2_, *S. enterica* Newport and *E. faecalis* cells were 45.2 and 42.8% of those measured by FCA, respectively. On the other hand, *E. coli* cells were more than 8-folds than that by FCA in the presence of 0.3 mg/ml TiO_2_.

**Table 2:** Bacterial species used in the study

**Species name**	**Gram**^**1**^	**Culture condition**	**Isolation**
*Salmonella enterica* serovar Newport	-	aerobic	human intestine
*Staphylococcus epidermidis* ATCC 12228	+	aerobic	human skin
*Enterococcus faecalis* ATCC 27274	+	anaerobic	human intestine
*Escherichia coli* ATCC 25922	-	anaerobic	human intestine

^1^+, Gram-positive; −, Gram-negative.

**Table 3:** Interference of oxide nanoparticles on bacterial quantification

**Nanoparticles (mg/ml)**	**ZnO**				**TiO**_**2**_				**SiO**_**2**_			
	***S. enterica*** **Newport (cells/ml)**^**a**^				***S. enterica*** **Newport (cells/ml)**				***S. enterica*** **Newport (cells/ml)**			
	**FMC**		**CFU**	**OD**_**660**_^**b**^	**FMC**		**CFU**	**OD**_**660**_	**FMC**		**CFU**	**OD**_**660**_
	**Total**	**Live**			**Total**	**Live**			**Total**	**Live**		
0	1.37 × 10^9^	1.36 × 10^9^	8.17 × 10^8^	1.37 × 10^9^	1.23 × 10^9^	1.22 × 10^9^	1.18 × 10^9^	1.23 × 10^9^	1.28 × 10^9^	1.26 × 10^9^	6.32 × 10^8^	1.28 × 10^9^
0.1	1.31 × 10^9^	1.30 × 10^9^	1.00 × 10^9^	1.46 × 10^9^	1.00 × 10^9^	9.94 × 10^8^	7.00 × 10^8^	9.16 × 10^8^	1.23 × 10^9^	1.22 × 10^9^	6.50 × 10^8^	1.20 × 10^9^
0.2	1.29 × 10^9^	1.28 × 10^9^	5.83 × 10^8^	1.28 × 10^9^	8.15 × 10^8^	8.05 × 10^8^	5.67 × 10^8^	5.89 × 10^8^	1.22 × 10^9^	1.20 × 10^9^	5.83 × 10^8^	1.18 × 10^9^
0.3	1.27 × 10^9^	1.14 × 10^9^	7.00 × 10^8^	1.19 × 10^9^	7.14 × 10^8^	7.06 × 10^8^	5.50 × 10^8^	3.23 × 10^8^	1.20 × 10^9^	1.18 × 10^9^	5.83 × 10^8^	1.16 × 10^9^
0.5	1.23 × 10^9^	1.21 × 10^9^	6.33 × 10^8^	1.01 × 10^9^	4.26 × 10^8^	4.13 × 10^8^	4.33 × 10^8^	-^c^	1.24 × 10^9^	1.21 × 10^9^	5.67 × 10^8^	1.15 × 10^9^
1	1.12 × 10^9^	1.10 × 10^9^	5.00 × 10^8^	7.15 × 10^8^	2.41 × 10^8^	2.35 × 10^8^	1.50 × 10^8^	-	1.22 × 10^9^	1.20 × 10^9^	7.17 × 10^8^	1.09 × 10^9^
	***S. epidermidis*** **ATCC 12228 (cells/ml)**				***S. epidermidis*** **ATCC 12228 (cells/ml)**				***S. epidermidis*** **ATCC 12228 (cells/ml)**			
0	3.53 × 10^8^	3.46 × 10^8^	9.33 × 10^7^	3.53 × 10^8^	4.46 × 10^8^	4.40 × 10^8^	1.20 × 10^8^	4.46 × 10^8^	5.20 × 10^8^	4.74 × 10^8^	2.00 × 10^8^	5.20 × 10^8^
0.1	2.13 × 10^8^	1.94 × 10^8^	2.18 × 10^7^	2.73 × 10^8^	1.21 × 10^8^	1.19 × 10^8^	2.00 × 10^7^	-	1.06 × 10^8^	9.57 × 10^7^	1.18 × 10^8^	4.48 × 10^8^
0.2	1.37 × 10^8^	1.18 × 10^8^	1.63 × 10^7^	1.23 × 10^8^	2.65 × 10^7^	2.62 × 10^7^	2.00 × 10^7^	-	7.27 × 10^7^	6.55 × 10^7^	6.50 × 10^7^	4.54 × 10^8^
0.3	1.71 × 10^7^	1.45 × 10^7^	1.37 × 10^7^	3.20 × 10^8^	1.46 × 10^7^	1.44 × 10^7^	3.33 × 10^7^	-	5.13 × 10^7^	4.60 × 10^7^	5.00 × 10^7^	5.00 × 10^8^
0.5	1.65 × 10^7^	1.45 × 10^7^	1.33 × 10^7^	1.85 × 10^8^	6.47 × 10^6^	6.40 × 10^6^	5.83 × 10^7^	-	6.72 × 10^7^	6.32 × 10^7^	5.83 × 10^7^	4.75 × 10^8^
1	3.31 × 10^7^	3.00 × 10^7^	1.10 × 10^7^	-	6.20 × 10^7^	6.11 × 10^7^	1.07 × 10^8^	-	2.21 × 10^8^	2.04 × 10^8^	1.18 × 10^8^	4.84 × 10^8^
	***E. faecalis*** **ATCC 27274 (cells/ml)**				***E. faecalis*** **ATCC 27274 (cells/ml)**				***E. faecalis*** **ATCC 27274 (cells/ml)**			
0	2.29 × 10^9^	2.28 × 10^9^	1.17 × 10^9^	2.29 × 10^9^	2.21 × 10^9^	2.17 × 10^9^	1.07 × 10^9^	2.21 × 10^9^	2.47 × 10^9^	2.42 × 10^9^	1.87 × 10^9^	2.47 × 10^9^
0.1	2.14 × 10^9^	2.06 × 10^9^	1.50 × 10^9^	2.13 × 10^9^	1.84 × 10^9^	1.81 × 10^9^	9.50 × 10^8^	1.69 × 10^9^	2.17 × 10^9^	2.11 × 10^9^	1.22 × 10^9^	2.40 × 10^9^
0.2	2.0 × 10^9^	1.92 × 10^9^	1.42 × 10^9^	1.73 × 10^9^	1.42 × 10^9^	1.40 × 10^9^	5.50 × 10^8^	1.07 × 10^9^	1.64 × 10^9^	1.61 × 10^9^	1.18 × 10^9^	2.30 × 10^9^
0.3	1.47 × 10^9^	1.44 × 10^9^	1.28 × 10^9^	1.54 × 10^9^	1.23 × 10^9^	1.22 × 10^9^	3.80 × 10^8^	5.26 × 10^8^	1.34 × 10^9^	1.33 × 10^9^	1.14 × 10^9^	2.23 × 10^9^
0.5	1.45 × 10^9^	1.40 × 10^9^	1.15 × 10^9^	8.57 × 10^8^	5.58 × 10^8^	5.54 × 10^8^	1.30 × 10^8^	-	8.69 × 10^8^	8.59 × 10^8^	7.00 × 10^8^	2.10 × 10^9^
1	1.07 × 10^9^	1.03 × 10^9^	7.00 × 10^8^	-	1.70 × 10^6^	1.60 × 10^6^	2.95 × 10^6^	-	4.44 × 10^8^	4.33 × 10^8^	5.00 × 10^8^	1.90 × 10^9^
	***E. coli*** **ATCC 25922 (cells/ml)**				***E. coli*** **ATCC 25922 (cells/ml)**				***E. coli*** **ATCC 25922 (cells/ml)**			
0	6.56 × 10^8^	5.64 × 10^8^	3.98 × 10^8^	6.65 × 10^8^	6.41 × 10^8^	6.32 × 10^8^	6.83 × 10^8^	6.41 × 10^8^	5.52 × 10^8^	5.46 × 10^8^	5.67 × 10^8^	5.52 × 10^8^
0.1	5.22 × 10^8^	4.95 × 10^8^	3.93 × 10^8^	6.18 × 10^8^	3.28 × 10^8^	3.26 × 10^8^	8.33 × 10^7^	4.86 × 10^8^	3.73 × 10^8^	3.68 × 10^8^	2.83 × 10^8^	5.21 × 10^8^
0.2	4.50 × 10^8^	4.17 × 10^8^	3.88 × 10^8^	5.56 × 10^8^	7.67 × 10^7^	7.61 × 10^7^	1.17 × 10^7^	3.07 × 10^8^	2.52 × 10^8^	2.49 × 10^8^	2.17 × 10^8^	5.08 × 10^8^
0.3	3.65 × 10^8^	3.54 × 10^8^	3.87 × 10^8^	4.97 × 10^8^	1.90 × 10^7^	1.88 × 10^7^	1.17 × 10^7^	1.63 × 10^8^	2.19 × 10^8^	2.16 × 10^8^	1.50 × 10^8^	5.11 × 10^8^
0.5	1.36 × 10^8^	1.17 × 10^8^	2.93 × 10^8^	2.89 × 10^8^	7.13 × 10^6^	6.97 × 10^6^	9.02 × 10^6^	-	2.03 × 10^8^	2.02 × 10^8^	2.50 × 10^8^	4.76 × 10^8^
1	1.43 × 10^8^	1.37 × 10^8^	3.10 × 10^8^	1.59 × 10^8^	2.21 × 10^7^	2.18 × 10^7^	4.58 × 10^7^	-	2.38 × 10^8^	2.37 × 10^8^	2.83 × 10^8^	4.67 × 10^8^

^a^Bacterial cell concentrations were measured by flow cytometry (FCM), culture-based counting for colony-forming units (CFU), and spectrophotometer method of optical density (OD) measurement after 1 hr exposure to different concentrations of ZnO, TiO_2_ and SiO_2_ nanoparticles; inoculum used for each experiment was indicated in the control samples, i.e. no nanoparticles.

^b^Presented data were converted from each sample cell concentration according to the each species standard curve of cell/ml *vs* OD_660_ and as mean of triplicate with standard deviations (SD) of < 5% from FCM and OD^600^ and <10% from CFU. Inoculum used for each experiment was indicated in the control samples, i.e. no nanoparticles.

^c^Value was negative.

### Effect of bacterial concentrations on quantification of bacteria after exposure to nanoparticles

In this experiment, we further investigated interference of nanoparticles ZnO (0.5 mg/ml), TiO_2_ (0.5 mg/ml), and SiO_2_ (1 mg/ml) on quantifications of *S. enterica* Newport, *S. epidermidis*, *E. faecalis*, and *E. coli* with various bacterial concentrations by comparison of flow cytometry with optical density measurement. As shown in Table 4, with the decreasing concentrations of the tested bacteria, no apparent interference of the nanoparticles on quantifications of all four bacterial species by flow cytometry was observed. However, with decreasing concentrations of the tested bacteria, the adverse effect on quantification of bacteria was dramatically increased using the spectrophotometer method of optical density measurement, which reflected a progressively worse estimate of the bacterial counts as the ratio of numbers of bacteria and nanoparticles in the suspension decreased. For example, in the presence of 0.1 mg TiO_2_, number of *S. enterica* Newport cells could not be detected due to high background interference from the nanoparticles in the samples. *S. enterica* Newport, *S. epidermidis*, *E. faecalis* could not be quantified in the presence of 0.5 mg/ml TiO_2_. The data obtained from the bacterial quantification in the presence of 0.5 mg/ ml of ZnO were either not able to be detected or not accurate. Due to lower interference of SiO_2_ at 1 mg/ml on the bacterial quantification, there was no apparent difference between flow cytometry and optical density measurement (Table 4).

**Table 4:** Quantification of bacterial cells at various concentrations in the presence of oxide nanoparticles

**Strain name**	**Control (No nanoparticles)**^**a**^			**ZnO (0.5 mg/ml)**			**TiO**_**2**_**(0.5 mg/ml)**			**SiO**_**2**_**(1 mg/ml)**		
	**FCM**		**OD**_**660**_^**b**^	**FCM**		**OD**_**660**_	**FCM**		**OD**_**660**_	**FCM**		**OD**_**660**_
	**Total cell no.**	**Live cell no.**		**Total cell no.**	**Live cell no.**		**Total cell no.**	**Live cell no.**		**Total cell no.**	**Live cell no.**	
*S. enterica* Newport	1.34 × 10^9^	1.31 × 10^9^	1.34 × 10^9^	1.20 × 10^9^	1.17 × 10^9^	7.47 × 10^8^	4.72 × 10^8^	4.63 × 10^8^	-	1.29 × 10^9^	1.29 × 10^9^	1.36 × 10^9^
	6.76 × 10^8^	6.61 × 10^8^	7.45 × 10^8^	5.18 × 10^8^	5.06 × 10^8^	1.73 × 10^8^	9.58 × 10^7^	9.21 × 10^7^	-	6.07 × 10^8^	6.06 × 10^8^	7.91 × 10^7^
	3.30 × 10^8^	3.20 × 10^8^	3.79 × 10^8^	2.19 × 10^8^	2.13 × 10^8^	-^c^	7.78 × 10^7^	7.34 × 10^7^	-	3.04 × 10^8^	3.03 × 10^8^	4.47 × 10^8^
	1.51 × 10^8^	1.47 × 10^8^	1.96 × 10^8^	8.89 × 10^7^	8.77 × 10^7^	-	6.56 × 10^7^	6.21 × 10^7^	-	1.19 × 10^8^	1.18 × 10^8^	2.87 × 10^8^
	1.18 × 10^8^	1.14 × 10^8^	1.50 × 10^8^	7.51 × 10^7^	7.37 × 10^7^	-	6.01 × 10^7^	5.68 × 10^7^	-	1.00 × 10^8^	9.99 × 10^7^	1.73 × 10^8^
*S. epidermidis*	3.43 × 10^8^	3.38 × 10^8^	3.43 × 10^8^	1.65 × 10^7^	1.50 × 10^7^	1.59 × 10^8^	3.06 × 10^7^	3.03 × 10^7^	-	1.75 × 10^8^	1.73 × 10^8^	3.96 × 10^8^
	1.73 × 10^8^	1.70 × 10^8^	1.59 × 10^8^	4.37 × 10^7^	3.66 × 10^7^	1.19 × 10^8^	6.91 × 10^7^	6.89 × 10^7^	-	1.57 × 10^8^	1.55 × 10^8^	1.59 × 10^8^
	8.41 × 10^7^	2.96 × 10^7^	6.67 × 10^7^	3.67 × 10^7^	2.94 × 10^7^	5.32 × 10^7^	5.34 × 10^7^	5.30 × 10^7^	-	7.56 × 10^7^	7.42 × 10^7^	7.96 × 10^7^
	4.10 × 10^7^	1.87 × 10^7^	2.69 × 10^7^	2.14 × 10^7^	1.63 × 10^7^	3.98 × 10^7^	2.88 × 10^7^	2.85 × 10^7^	-	3.57 × 10^7^	3.48 × 10^7^	2.69 × 10^7^
	4.04 × 10^7^	1.48 × 10^7^	1.37 × 10^7^	1.74 × 10^7^	1.32 × 10^7^	2.69 × 10^7^	3.27 × 10^7^	3.25 × 10^7^	0	3.99 × 10^7^	3.87 × 10^7^	2.69 × 10^7^
*E. faecalis*	2.33 × 10^9^	2.32 × 10^9^	2.33 × 10^9^	1.20 × 10^9^	1.16 × 10^9^	8.82 × 10^8^	5.54 × 10^8^	5.33 × 10^8^	-	7.10 × 10^8^	7.07 × 10^8^	2.02 × 10^9^
	1.20 × 10^9^	1.19 × 10^9^	1.27 × 10^9^	1.44 × 10^8^	1.26 × 10^8^	-	8.43 × 10^6^	8.10 × 10^6^	-	3.17 × 10^8^	3.14 × 10^8^	1.41 × 10^9^
	5.86 × 10^8^	5.68 × 10^8^	5.94 × 10^8^	4.26 × 10^7^	4.00 × 10^7^	-	4.30 × 10^6^	4.20 × 10^6^	-	1.43 × 10^8^	1.42 × 10^8^	5.94 × 10^8^
	2.78 × 10^8^	2.74 × 10^8^	2.60 × 10^8^	4.00 × 10^7^	3.87 × 10^7^	-	2.02 × 10^7^	1.98 × 10^7^	-	1.20 × 10^8^	1.17 × 10^8^	3.37 × 10^8^
	2.27 × 10^8^	2.21 × 10^8^	2.08 × 10^8^	3.62 × 10^7^	3.53 × 10^7^	-	2.52 × 10^7^	2.48 × 10^7^	-	1.16 × 10^8^	1.13 × 10^8^	2.86 × 10^8^
*E. coli*	6.04 × 10^8^	5.57 × 10^8^	6.04 × 10^8^	8.96 × 10^7^	7.17 × 10^7^	2.94 × 10^8^	1.69 × 10^7^	1.50 × 10^7^	-	2.17 × 10^8^	2.04 × 10^8^	5.51 × 10^8^
	2.98 × 10^8^	2.76 × 10^8^	3.21 × 10^8^	6.04 × 10^7^	4.17 × 10^7^	9.85 × 10^7^	4.89 × 10^7^	4.39 × 10^7^	-	2.07 × 10^8^	1.93 × 10^8^	3.38 × 10^8^
	1.51 × 10^8^	1.41 × 10^8^	1.52 × 10^8^	4.80 × 10^7^	3.42 × 10^7^	-	5.99 × 10^7^	5.11 × 10^7^	-	1.38 × 10^8^	1.23 × 10^8^	1.87 × 10^8^
	6.55 × 10^7^	6.02 × 10^7^	6.34 × 10^7^	3.75 × 10^7^	2.51 × 10^7^	-	5.12 × 10^7^	4.20 × 10^7^	-	6.31 × 10^7^	5.55 × 10^7^	8.11 × 10^7^
	5.47 × 10^7^	5.20 × 10^7^	3.68 × 10^7^	3.28 × 10^7^	1.87 × 10^7^	-	4.47 × 10^7^	4.07 × 10^7^	-	5.10 × 10^7^	4.44 × 10^7^	8.11 × 10^7^

^a^Bacterial cell number was measured by flow cytometry (FCM) and spectrophotometer method of optical density (OD) measurement after 1 hr exposure to ZnO, TiO_2_ and SiO_2_ nanoparticles; inoculum used for each experiment was indicated in the control samples, i.e. no nanoparticles.

^b^Presented data were converted from each sample cells concentration according to the each species standard curve of cell/ml *vs* OD_660_ and as mean of triplicate with standard deviations (SD) of < 5%.

^c^Value was negative.

## Conclusions

In summary, this study compared three most commonly used bacterial quantification methods including colony counts, spectrophotometer method of optical density measurement, and flow cytometry in the presence of metal oxide nanoparticles. Our results demonstrated that flow cytometry is the best method with no apparent interference by the nanoparticles, indicating that it is suitable for rapid, accurate and automatic detection of bacteria. Flow cytometry is also able to detect both live and dead bacterial cells and allows detection of all bacteria including those that are uncultured. Although the bacterial quantification determined by plate counts was not affected by the nanoparticles, it was time consuming, less accurate and not suitable for automation. The spectrophotometer method using optical density measurement was the most unreliable method to quantify and detect bacteria in the presence of oxide nanoparticles. The data presented in this study indicated that flow cytometry method for bacterial quantification is superior to the other two methods. This study provides data examining the potential interference of oxide nanoparticles on bacterial quantification. The information provided here will be useful in the assessment of bacterial contamination in food, drug and cosmetic products containing nanoparticles. Future studies on other nanoparticles and limit of the bacterial detection by FMC are warranted.

## Methods

### Materials and preparation of nanoparticle suspensions

ZnO (purity >97%), TiO_2_ (purity ≥99.5%), and SiO_2_ (purity 99.5%) nanoparticles were purchased from Sigma Chemical Co. The LIVE/DEAD BacLight bacterial viability and counting kit containing solutions of 3.34 mM SYTO9 in dimethyl sulfoside (DMSO, 200 μl), 20 mM propidium iodide (PI) in DMSO (200 μl) and a calibrated suspension of microspheres (diameter: 6 μm, 1 ml; concentration: 1.0 × 10^8^ beads/ml) and SYTO 9 green fluorescent nucleic acid stain (5 mM solution in DMSO, 100 μl) were purchased from Molecular Probes (Eugene, Oregon). Suspensions of the nanoparticles were prepared with Milli-Q water by means of ultrasonic vibration in a BRANSON 3200 UltraSonic Cleaner for 30 min and the stock solutions were vortexed briefly before each use [40]-[42].

### Physical and chemical characterizations of nanomaterials

The size, shape and morphology of ZnO, TiO_2_ or SiO_2_ nanoparticles were determined using transmission electron microscopy (TEM). The nanoparticles were homogeneously dispersed in Milli-Q water, and 3 μL suspensions was deposited on the TEM grid, dried, and evacuated before analysis. Images were collected using a field emission JEM-2100 F (JEOL, Tokyo, Japan) equipped with a CCD camera in high resolution mode with an acceleration voltage of 100 kV.

The hydrodynamic size and zeta potential were measured in Milli-Q water using a Zetasizer (Malvern, Worcestershire, UK) as described in previous study [43]. Briefly, the nanoparticle samples were measured after dilution of a nanoparticle stock solution to 50 μg/ml in Milli-Q water. These dilutions were sonicated for 30 min and vortexed briefly to provide a homogenous dispersion. For the size measurement, 70 μL of the diluted dispersion nanoparticles was transferred to a cuvette for dynamic size measurement; for zeta potential measurement, a Malvern zeta potential cell was washed three times with ultrapure water followed by transferring 850 μl of diluted dispersion nanoparticles to this cell to measure the zeta potential. The concentration of the samples and experimental methods were optimized to assure the quality of the data. NIST standard gold nanoparticles (10 nm, 30 nm, and 60 nm) were used in the validation of the instrument. Both size and zeta potential were measured at least three times. The data were calculated as the average size or zeta potential of nanoparticles.

### Bacterial strains and culture conditions

Four bacterial species were chosen for all experiments (Table 2). The bacterial stock cultures were stored in freezer (−80°C) with glycerol to a final concentration of 15%. *E. faecalis* and *E. coli* from the glycerol stocks were streaked into brain heart infusion (BHI) agar plates at 37°C overnight in an anaerobic chamber (Coy Laboratory Products INC.). For *S. enterica* Newport and *S. epidermidis*, the plates were grown under aerobic condition. One colony was picked by a loop and inoculated into a 50-ml Falcon centrifuge tube containing 10 ml BHI medium. The cultures were incubated anaerobically or aerobically in static conditions at 37°C overnight for use as seed cultures. Each seed culture of the bacteria was inoculated into BHI medium with an inoculation ratio of 1% (v/v), except *S. epidermidis* with 10% (v/v) of inoculation into BHI medium, and then the cultures were incubated at 37°C under anaerobic or aerobic condition without agitation for 4–6 hrs to enter the exponential phase based on our preliminary experiments [40]. The cells were collected by centrifugation (10,000 rpm, 2 min, 4°C), washed twice and then re-suspended in sterile saline solution (0.85% NaCl), which served as experimental bacterial cells. To measure the bacterial numbers in the presence of different concentrations of nano ZnO, TiO_2_, and SiO_2_, various concentrations of the nanoparticles (final concentrations were 0, 0.1, 0.2, 0.3, 0.5, 1 mg/ml) based on our based on our preliminary experiments were added into each bacterial cell re-suspension and mixed well by vortexing, leaving one as a control without nanoparticles, but same volume of Milli-Q water, and then kept in the dark for 1 hr at 4°C [39]. In order to test the different bacterial concentrations after exposure to the nanoparticles, the initial bacterial re-suspension with approximately 10^8^-10^9^ cells/ml was serially diluted (0, −2, −4, −8, −10 fold) in saline solution and then mixed well with each nanoparticles at final concentration of 0.5 mg/ml, 0.5 mg/ml and 1 mg/ml for ZnO, TiO_2_ and SiO_2_, respectively. All the samples were kept under the same conditions as mentioned above. A control (containing saline solution and nanoparticles without bacterial cells) was included in all experiments and kept under same conditions. All experiments were carried out in triplicates. After 1 hr exposure to the nanoparticles, the bacterial cell concentrations were measured by different methods as mentioned below [40],[41],[44],[45].

### Plate counting cell numbers

Samples were withdrawn and then serially diluted in saline solution. Aliquots of 10 μl were spread on BHI-plates. After overnight incubation at 37°C, colonies on the plates were counted to determine the number of CFU [46].

### Optical density measurement

Aliquots of 200 μl were withdrawn, added to a 96-well plate (Corning incorporated, flat bottom, non-lid) and immediately assayed by measuring the optical density in a SpectraMax M2 plate reader (Molecular Devices) at 660 nm [41]. The absorbance values of the controls were subtracted from the experimental values [36].

### Flow cytometry analysis of bacterial cell numbers in combination with LIVE/DEAD BacLight bacterial viability and counting kit

Samples were collected, diluted and stained according to the manufacture’s instruction using the BacLight LIVE/DEAD bacterial viability and counting kit as described briefly here. Each of 1.5 μl of 3.34 mM SYTO 9 green fluorescent nucleic acid dye (Component A) and of 20 mM propidium iodide (Component B) was added to the flow cytometry tube containing 1 ml sample. Incubate the sample for 15 minutes at room temperature protected from light. The bacterial cell numbers assay was performed on the Accuri C6 flow cytometry. Fluorescence filters and detectors were all standardized with green fluorescence collected in the FL1 channel (530 ± 15 nm) and red fluorescence collected in the FL3 channel (>670 nm). All parameters were collected as logarithmic signals. A similar setup of parameters was used as described previously [40]. Data were analyzed using CFlow Plus software. In density plots of light scatter properties, bacterial cells were gated from irrelevant counts for fluorescence analyses. In density plots of fluorescence, the distinct bacterial populations (live cells and damaged or dead cells) were gated based on the different viability stages. Total cell numbers = live cell numbers + dead cell numbers. Accuri C6 flow cytometry was calibrated using 8-peak Spherotech Validation Beads m.

### Standard curve of optical density versus cell number for each bacterial stain

**Figure 3:**
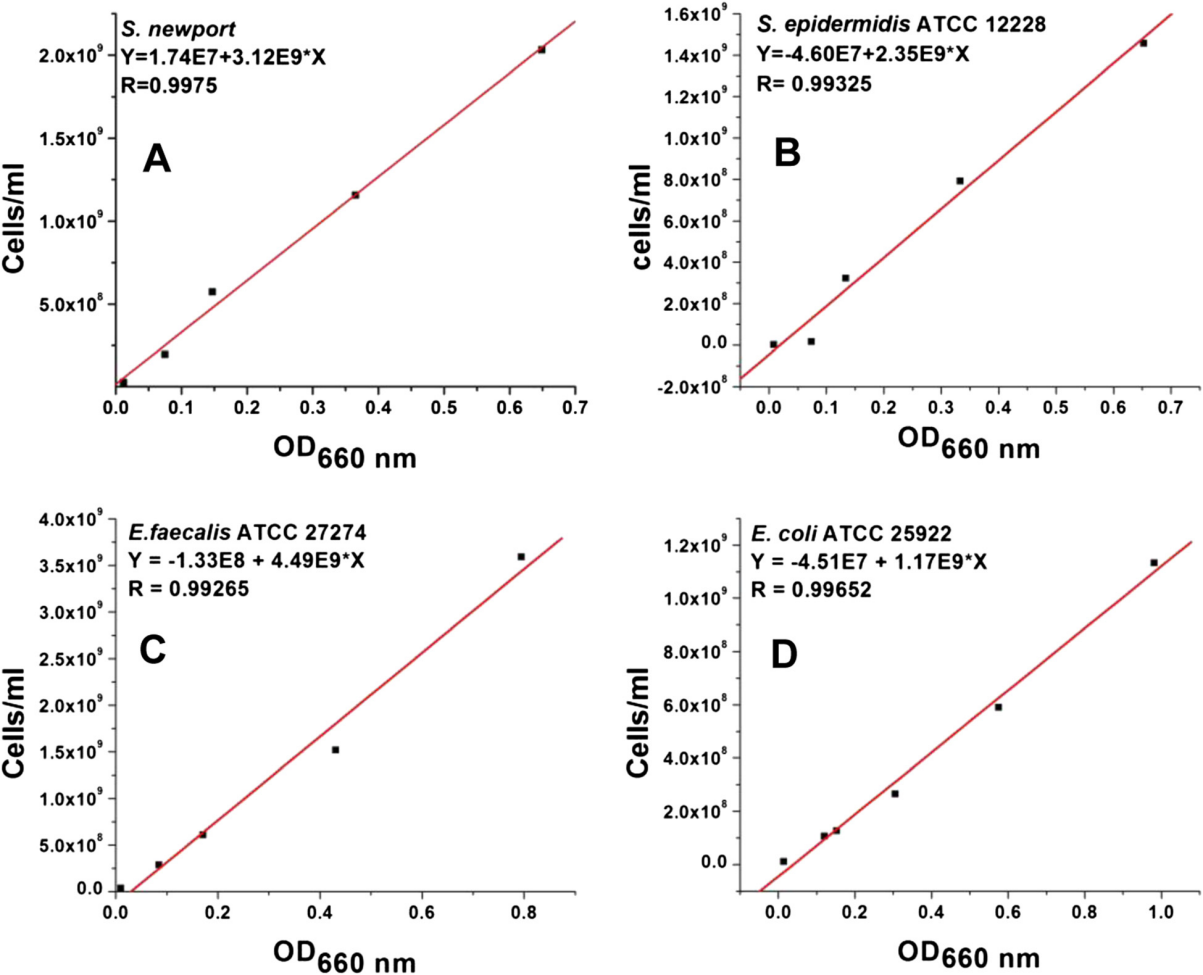
**Standard curve of optical density (OD) versus bacterial cell number obtained by flow cytometry (FCM) containing no nanoparticles. A**, S. enterica Newport; **B**, S. epidermidis; **C**, E. faecalis; **D**, E. coli. The correlation between OD660 and bacterial cell number for each species was established by means of a standard curve. Data are presented as mean of triplicate with standard deviations (SD) of < 5%. Y is cells/ml; X is OD660 nm value; E is 10^; R is correlation coefficient.

Exponentially growing cells of each bacterial species were serially diluted in saline solution in triplicate. Then OD_660_ of the samples was measured by above mentioned method. Sterile saline solution was used as blanks. For counting cell numbers, the serially diluted bacterial cultures were further diluted to 1 ml with saline solution. Then the total bacterial cell number was analyzed by flow cytometry as mentioned above. The correlation between OD_660_ and cells number for each bacterial species was established by means of a standard curve (Figure 3).

## Competing interests

The authors declare that they have no competing interests.

## Authors’ contributions

HP, YZ, GH, NK, and HC performed research and analyzed data. HC conceived and designed the project. HC wrote the paper with help from all authors. The final manuscript was read and approved by all authors.
